# Asymmetry in the brain influenced the neurological deficits and infarction volume following the middle cerebral artery occlusion in rats

**DOI:** 10.1186/1744-9081-4-57

**Published:** 2008-12-22

**Authors:** Huanmin Gao, Meizeng Zhang

**Affiliations:** 1 Department of Neurology, the Second Affiliated Hospital of Qingdao University Medical College, Qingdao 266042, PR China; 2 Department of Neurology, Affiliated Hospital of Qingdao University Medical College, 16 Jiang Su Road, Qingdao 266003, PR China

## Abstract

**Background:**

Paw preference in rats is similar to human handedness, which may result from dominant hemisphere of rat brain. However, given that lateralization is the uniqueness of the humans, many researchers neglect the differences between the left and right hemispheres when selecting the middle cerebral artery occlusion (MCAO) in rats. The aim of this study was to evaluate the effect of ischemia in the dominant hemisphere on neurobehavioral function and on the cerebral infarction volume following MCAO in rats.

**Methods:**

The right-handed male Sprague-Dawley rats asserted by the quadrupedal food-reaching test were subjected to 2 hours MCA occlusion and then reperfusion.

**Results:**

The neurological scores were significantly worse in the left MCAO group than that in the right MCAO group at 1 h, 24 h, 48 h and 72 h (*p *<*0.05 *respectively). There was a trend toward better neurobehavioral function recovery in the right MCAO group than in the left MCAO group. The total infarct volume in left MCAO was significantly larger than that in the right (*p *< 0.05).

**Conclusion:**

The neurobehavioral function result and the pathological result were consistent with the hypothesis that paw preference in rats is similar to human handedness, and suggested that ischemia in dominant hemisphere caused more significant neurobehavioral consequence than in another hemisphere following MCAO in adult rats. Asymmetry in rat brain should be considered other than being neglected in choice of rat MCAO model.

## Background

Focal cerebral ischemia models in rats have gained increasing acceptance in recent years for their relevance to human beings [1,2]. But rat brain injury produced by MCAO varies considerably in its size and distribution. The sides picked for MCA occlusion is also different between laboratory studies. However, little is really recognized about the difference between the right MCAO and the left MCAO in rat.

It had been established that lateralization is the uniqueness of the humans [3-5]. However, this concept has been challenged. That paw preference in rats is similar to human handedness has been known for decades, as stated by Rogers "... lateralization in humans is not unique either in nature or extent [6]." However, it has been less well recognized that lateralization of paw in rats may result from dominant hemisphere of rat brain. Greater ischemic severity in dominant hemisphere may be even more controversial. We indeed suspected that the left and the right MCAO rat models might be different, in that the two hemispheres in the rat are not the same entirely. Therefore, the present study was designed to investigate whether there is a greater stroke severity in the dominant hemisphere in transient focal ischemia rat model.

## Methods

### The paw preference determination

**Figure 1:**
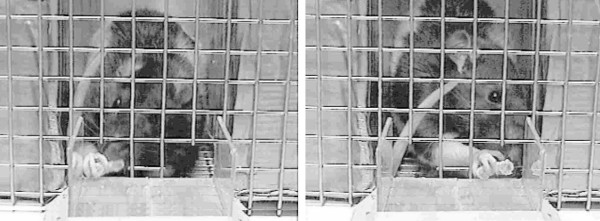
**Schematic diagram showing the modified quadrupedal food-reaching test originally developed by Tang and Verstynen (2002)**.

The Animal Subjects Committee of Qingdao University Medical College approved this protocol. A modified quadrupedal food-reaching test developed by Tang and Verstynen [7] has been used previously (Figure 1) to assess the paw preference in male Spraque-Dawley rats (Department of Experimental Animals, Chinese Academy of Science, Shanghai, China) weighting 260~280 g [8]. Briefly, rats adapted to the animal room for one week were deprived of food for two days and then individually placed in a metal housing cage with two front openings separated by 1 cm in the day of paw preference test. The openings in the testing cage were small enough to allow access to food by a forepaw only, not by the snout. An observer scored the numbers of right and left paw reaches. Rats were classified as right-hander, left-hander, and mixed-hander based on the binomial probability distribution test [9]: rats were considered as right-handed if the right-paw entry (RPE) score was equal to or greater than 29, left-handed when the score was equal to or smaller than 21 and ambidextrous when the RPE score was between 22 and 28.

### Middle cerebral artery occlusion (MCAO) model

The transient focal cerebral ischemia rat model has been described by Longa EZ *et al *previously [10]. Briefly, 24 right-handed male Sprague-Dawley rats fasted overnight were randomized to two groups: the right MCAO group: sham (n = 3), ischemia (n = 9); and the left MCAO group: sham (n = 3), Ischemia (n = 9). The rats were anesthetized with 8% chloral hydrate (300 mg/kg, *i.p*.) and were positioned supine. Under an operating microscope, the right (or left in the second group) common, internal, and external carotid arteries were exposed through a para-median incision of the neck. The external carotid artery was ligated. A 4-0 nylon surgical thread with round tip coated with poly-L-lysine was inserted about 18–20 mm through external carotid artery until the distal end met mild resistance, indicating the occlusion of the origin of the middle cerebral artery (MCA). The suture was inserted into the corresponding MCA in each group.

After 2 h occlusion, reperfusion was instituted by withdrawing intraluminal suture. The sham-operated animals were treated identically but the MCA was not occluded.

Rectal temperature was continuously monitored and maintained at approximately 37°C with heat lamps and a heat pad. After restoration of blood flow, the animals were allowed to recover at ambient temperature (25°C, by an air-conditioner). The efficiency of MCAO was determined by the neurological assessment according to the modified method described by Bederson *at al *[11].

### Neurological deficits assessment

Within the 72-h observation period, behavioral tests were performed daily in all 24 rats before and during MCAO by an investigator who was blinded to the experimental groups. The tests have been used previously to evaluate various aspects of neurological function: the postural reflex test developed by Bederson *at al *[11] to examine upper body posture while the animal is suspended by the tail. In the dysfunctional paw test, the contra lateral forepaw or hind paw was pulled toward the body, and the time to re-extend each paw was scored as: 0 (< 1 second), 1 (< 5 seconds), or 2 (> 5 seconds). In the postural reflex test, the rat was pushed in the contra lateral direction and scored as: 0 (resistance to lateral push), 1 (initially reduced but progressive resistance), 2 (reduced resistance), or 3 (lateral down fall). In the circling test, movements were scored as: 0 (straight movement), 1 (movement to the right), 2 (circling movement), or 3 (no movement). Each score was summed and represented as a single overall neurological score (0 to 10).

### Brain infarct assessment

Rats were allowed to survive for 72 h. After decapitation, the brains were immediately removed, post-fixed for 2 h in 20% sucrose in 4% paraformaldehyde and kept in 30% sucrose in 0.01 mol/L PBS until they sunk to the bottom. Coronal sections (30 μm) were cut on a freezing microtome and kept in cryoprotective solution at -20°C. Brain sections were stained with hematoxylin and eosin (H&E). To quantity brain infarct volume and depict infarct frequency distribution, coronal sections were viewed using Leica Q500 IW image processing system (Leica, Bensheim, Germany). The volume of cerebral infarction was calculated as the product of cross-sectional area for all sections and distance between sections, using the formula:

***V = t(A1+A2+...+An) - t(A1+An)/2***

V: cerebral infarction volume (mm^3^);

t: 30μ (distance between neighbor slices);

*A: the cerebral infarction area at each slice*.

### Statistical analysis

Values are presented as mean ± SD. Differences between groups were analyzed with Student's two-tailed *t*-test. In the behavior test, comparison of groups was made by Mann-Whitney *U *test. α level was set to 0.05 to determine statistical significance.

## Results

### Neurological deficits score comparisons

**Figure 2:**
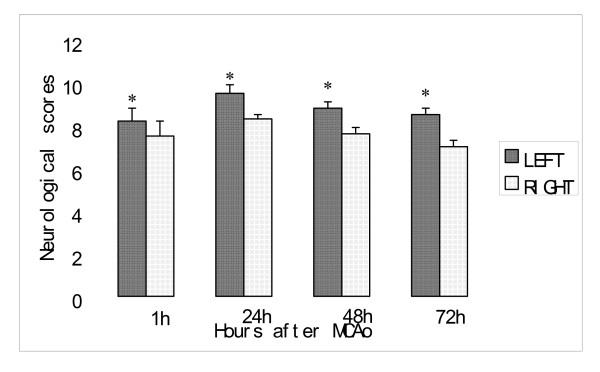
**Comparison of total neurological scores at various times after 2 hours of MCAo in rats with left MCA suture and right MCA sutures**. Normal neurological deficit scores are 0; maximal score is 10.

Neurological deficits were observed in all rats. All animals exhibited impairment of postural reflexes. Contralateral forelimb placing deficits were clearly present at 50 minutes following MCAO. The neurological deficits scores after 2 h MCAO were significantly worse in the left MCAO group than that in the right MCAO at 1 h, 24 h, 48 h and 72 h (8.2 ± 0.6 versus 7.5 ± 0.7, 9.5 ± 0.4 versus 8.3 ± 0.2, 8.8 ± 0.3 versus 7.6 ± 0.3, and 8.5 ± 0.3 versus 7.0 ± 0.3, p < 0.05 respectively). There was a trend toward better neurological function recovery in the right MCAO than in the left MCAO group (Figure 2). The result showed that lesions in dominant hemisphere produced a more severe and prolonged neurological deficit.

### Assessment of brain damage

**Figure 3:**
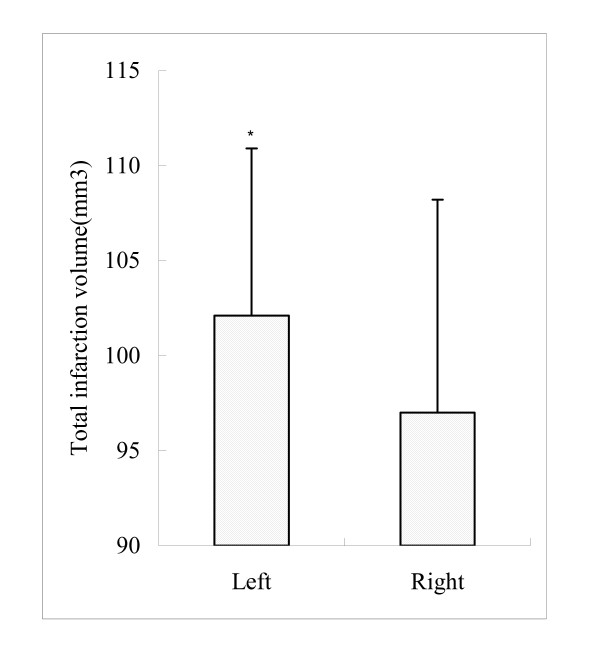
**Comparison of total infarct volumes in left MCA sutures and right MCA sutures groups 72 h after MCAO in rats**.

Examination of the areas of cerebral infarction revealed pancellular necrosis as well as dense areas of eosinophilic, shrunken neurons along the edges of the infarct. An extensive brain infarct was found in the dorsolateral and lateral portions of neocortex and the entire caudoputamen in rat brains. Smaller areas of cortical infarct were found in rat with right MCAO compared with left MCAO. With 2 h MCAO, the total brain infarct volume was significantly larger in rats with left MCAO than that in the right MCAO group (Figure 3, 102.1 ± 8.8 mm^3 ^versus 97.0 ± 11.2 mm^3^; *p *= 0.04 < 0.05). This result suggested that the volume of cerebral infarction in the dominant hemisphere was larger than that in the non-dominant hemisphere in adult rats.

## Discussion

It has been extensively believed that handiness is one of the prominent markers of functional asymmetry of human brain. A lateralized population means that more than 50% of the individuals are lateralized in the same direction. It is true that approximately 90% of human population shows a right hand preference in the United States [12]. However in population-level, several lines of evidences have demonstrated that lateralization in humans is not unique either in nature or extent. There is now accumulating evidence for population-level asymmetries in animals including rats and other rodent animals [13]. In rats, population-level right-handedness was reported early in1930. The distribution of hand preference in rats is similar to human hand preference. Therefore the uniqueness of men in population-level right-handedness is rejects [7]. On the contrary, a lot of research reports especially in the relatively old literature indicated no population-level right-handedness in the rat. These seemingly inconsistent results in the literature can be explained in the terms of the differences among testing methods according to Tang and Verstynen [7].

The result of paw preference in rats using the modified computerized food-reaching test by Tang and Verstynen [7] showed that 99.5% of the right handed rats first used their right paw to reach the food, meanwhile only 0.5% of these using the left paw to reach the food. Similar to the right handed ones, 98.6% of the left-handed rats first used their left paw to reach the food, meanwhile only 1.4% first used their right paw in food reaching. In the present study we selected the right-handed rats using the modified food-reaching test and found that about 80% of rats were right-handed and nearly 20% of rats were left-handed. In order to get enough number of rats for the present study we chose the majority (the right-handed adult rats). Also we used only male adult rats to avoid the estrogenic hormones interferences on the paw preference and cerebral infarction.

The asymmetry in the rat brain also influenced the chemical asymmetry including dopamine and norepinephrine in normal adult rats or in the rat with cerebral infarction involving cortex and of frontal cortex in particular [14]. As expected, hand preference in rats is controlled by the contralateral primary motor cortex, since handiness was reversed after ablation of this region [15]. In the right-handed rats the left hemisphere could play a more important role in the process of new visual-motor learning [16]. Regarding the role of left-brain in cognitive function, it was also shown that the latency of visual evoked responses was significantly shorter from the left brain than the right brain [17]. The asymmetric cognitive control in an animal model may have a major impact in many aspects of biology in respect to normal functioning, superior talents, and diseases. The insular cortex is involved in almost half of patients with nonlacunar ischemic MCA territory strokes. Major insular involvement is associated with large MCA territory infarcts, proximal MCA occlusions, and greater stroke severity [18]. The right-handed rats with their left hemisphere of the brain as the dominant hemisphere were selected in the present study. The total cerebral infarction volume produced by the left MCAO was larger than that in the right MCAO, and a significantly more severe and prolonged neurological deficit was demonstrated in adult rats following MCAO. So our present study was consistent with the concept of asymmetry of rat brain on the neurological function and pathological observation following MCAO.

Therefore it might be speculated that it is urgent to pay more attention to the asymmetry in the rat brain when using the MCAO rat model. The limitation of the present study includes the number of animals used in each group, and therefore the inability to perform more extensive statistical analysis of all variables such as body weight in terms of their relations with cerebral infarction volumes. It is hoped that further studies will be directed toward this goal.

## Conclusion

Our experiments using neurological behavioral function evaluation and brain pathological study showed that cerebral infarction in dominant hemisphere produced larger brain infarct volume and a more severe and prolonged neurological deficit significantly in adult rats following MCAO. The present result was consistent with the hypothesis that paw preference in rats is similar to human handedness. Asymmetry in rat brain should be considered other than being neglected in choice of rat MCAO model.

## List of abbreviations used

MCAO: the middle cerebral artery occlusion; MCA: the middle cerebral artery; RPE: right-paw entry; H&E: hematoxylin and eosin.

## Competing interests

The authors declare that they have no competing interests.

## Authors' contributions

ZM and GH were responsible for design and writing of the manuscript. GH carried out the data acquisition and analyses. Both authors read and approved the final manuscript.
